# The coexistence of hypercalcemia and hypoglycemia in a patient with a renal tumor and B cell lymphoma

**DOI:** 10.1590/2359-3997000000212

**Published:** 2016-09-26

**Authors:** Jimena Soutelo, Sofía Moldes, Cielo Frisone, Laura Salvá, Cecilia Agostinis, Gabriel Faraj

**Affiliations:** 1 Hospital Churruca Buenos Aires Argentina Servicio de Endocrinología, Hospital Churruca, Visca, Buenos Aires, Argentina

## Abstract

Paraneoplastic syndromes are a heterogeneous group of malignant diseases caused by events which involve endocrine, immune and metabolic aspects and whose symptoms vary according to the substance produced and the primary tumor. Hypercalcemia is a frequent complication in cancer patients. Prognosis of cancer patients with hypercalcemia is usually poor. A factor called parathyroid hormone related peptide, whose actions are similar to those of the parathyroid hormone, is thought to be the most common cause of malignancy associated hypercalcemia. Non-islet hypoglycemic cell tumor consists of a rare syndrome characterized by the presence of a solid tumor and severe fasting hypoglycemia determined by an insulin-independent pathway. We report a case of a 59-year-old-man with a renal tumor and a T-cell rich large B cell lymphoma who was hospitalized due to severe hypercalcemia and hypoglycemia. The laboratory examination reported hypercalcemia with inhibited PTH and hypoglycemia with inhibited insulin secretion, arriving to the conclusion of tumoral peptide production. He received denosumab and corticoid therapy. The patient died one month later despite initial improvement after medical treatment. While a single paraneoplastic manifestation may be expected in most tumors, the coexistence of two or more of them is rare, except in hepatocellular carcinomas, and it has not yet been described in renal tumors.

## INTRODUCTION

Tumors usually produce symptoms by invasion, obstruction and bulk mass on the primary localization of neoplasm and their regional or distant metastases. In addition, tumors can produce signs at a distance from their localization. These are the so-called paraneoplastic syndromes (PNS) and are caused by substances produced by tumor and distributed by circulation to act on target organs. Most of these substances are polypeptide hormones, autoantibodies, growth factors, cytokines, hormones and their precursors ( 1 ).

PNS presents as an heterogeneous group of manifestations (cutaneous, neurological, endocrine, hematologic, rheumatological or renal) which involve endocrine, immune and metabolic aspects, and whose symptoms vary according to the produced substance and the original tumor. The suspicion of these manifestations – which depends on the type of tumor and may preceed the diagnosis of cancer – enables the diagnosis of the oncological pathology even in earlier stages. The successful treatment of the underlying disease leads to the improvement of the PNS ( 2 ).

The most common endocrine manifestations are the inappropriate antidiuretic hormone secretion, Cushing’s syndrome, hypercalcemia and hypoglycemia as well as hypocalcemia, osteomalacia, hypercholesterolemia and hyperuricemia ( 2 ).

Hypercalcemia is a common complication in cancer patients and has been reported to occur in up to 20 to 30 percent of patients with cancer and in almost 100 percent of patients with multiple myeloma. Detection of hypercalcemia in an oncological patient is a poor prognosis factor.

The main factor responsible for malignant hypercalcemia is the PTHrP, whose actions are similar to PTH and regulates bone resorption and renal handling of calcium and phosphate ( 3 ).

The tumors that more frequently cause hypoglycemia are mesenchymal, hepatocellular, gastrointestinal, lymphomas and adrenal carcinomas. Tumor associated hypoglycemia may be caused by a non-suppressible production of IGF-1, IGF-2, hypermetabolism of glucose, tumor cell production of insulin, insulin binding to a monoclonal protein or insulin receptors proliferation ( 4 ).

In this case report we describe the coexistence of two paraneoplastic manifestations – hypoglycemia and hypercalcemia – in a patient with a renal tumor and B cell lymphoma.

## CASE REPORT

A 59-year-old male patient with a medical history of tobacco smoking, Chronic Kidney Disease (CKD) stage IV ( *Kidney Disease Outcome Quality Initiative* ) and T-cell histiocyte-rich large B-cell lymphoma diagnosed in May 2014. The patient underwent six courses of chemotherapy consisting of rituximab, cyclophosphamide, doxorubicin hydrochloride, vincristine and prednisolone with partial response.

During hematology controls, a PET scan was performed which revealed an hypermetabolic mass of 56 X 58 mm in the anterior valve of the right kidney Standard uptake value (SUV) 5.2, which was in staging studies. As it did not improve after chemotherapy treatment, primary renal neoplasia was suspected and therefore the department of urology prescribed a nephrectomy. The patient refused to undergo surgery.

In June 2015, he was admitted to our hospital with asthenia, dehydration and a general bad clinical condition. His physical examination revealed palpable lymph nodes with a painless hard-stone one in the left groin with ipsilateral lower limb lymphedema. Laboratory results revealed worsened renal function and hypercalcemia.

During hospitalization, he was performed routine laboratory tests, his level of phosphorus and calcium were evaluated as well as his thyroid function ( Table 1 ). Malignant hypercalcemia was diagnosed, and treated with isotonic saline hydration and 120 mg denosumab subcutaneous injection. Given the patient’s impaired renal function, oral vitamin D supplementation was used to avoid unwanted hypocalcaemia ( 5 ). Serum calcium levels decreased ( Graphic 1 ). Daily glucose controls showed asymptomatic fasting hypoglycemia. Adrenal insufficiency and pharmacological causes of hypoglycemia were ruled out. Episodes of hypoglycemia persisted in despite of the improvement in the glomerular filtration rate. Laboratory workup was performed to determine its etiology ( Table 1 ). Fasting hypoglycemia with hypoinsulinemia and decreased growth hormone (GH), IGF-1 and insulin growth factor binding protein 3 (IGFBP3) were found.

**Table 1:** Laboratory tests

Test	Values	Reference values
Calcium	14.12	8.5-10.5 mEq
Phosphorus	3.9	2.5-4.5 mEq
Albumin	3.6	3.5-5 g/dL
Cortisol	18.67	5-25 ug/dL
25-hydroxyvitamin D	8	> 30 ng/mL
PTH	3	11-67 pg/mL
TSH	1.8	0.5-4 uUI/mL
T4	8	4.5-12 ug/dL
FT4	1.21	0.6-2 ng/dL
T3	53	60-220 ng/dL
Glycemia	67	70-100 mg/dL
Insulin	2.1	h/18.7 uUI/mL
Insulin/glycemia	0.031	< 0.3
C Peptide	2.03	0.8-4 ng/mL
GH	1.36	h/3 ng/mL
IGF-1	< 25	75-240 ng/mL
IGFBP3	0.9	0.9-3.7 mg/mL

PTH: parathormone; TSH: thyrotropin; T4: thyroxine; FT4: free thyroxine; T3: triiodothyronine; GH: growth hormone; IGF-1: insulin-like growth factor 1; IGFBP3: binding protein insulin-like growth factor.

**Graph 1:**
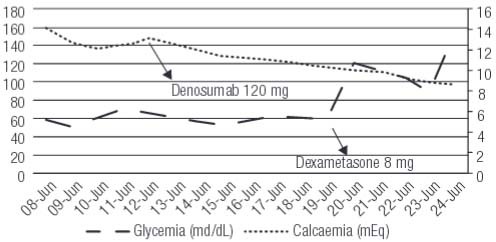
Clinical course of hypercalcemia (black dots) and hypoglycemia (black stripes). Hypercalcemia begins with a value of 14.12 mg/dL, and decreases after denosumab was administered, with an excellent response. Hypoglycemia stays below values of 60 mg/dL, and increases after dexamethasone administration.

**Figure 1:**
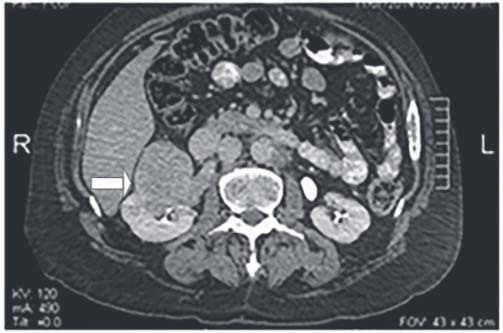
CT, where a solid formation of 53 x 56 mm is observed in the right kidney with microcalcifications. Cortical atrophy and a decrease in its size, with ureteropelvic dilatation of 20 mm.

A chest, abdomen and pelvis tomography (TC) showed moderate left pleural effusion and a 53 x 56 mm solid mass with microcalcifications in the right kidney, which produced cortical atrophy, reduction of the kidney’s size and a 20 mm ureteropelvic dilatation ( Figure 1 ). These characteristics added up to the hypermetabolic activity of the renal mass were interpreted as malignant tumor. At the retroperitoneal and mesenteric territory, lymphadenopathy of significant size, compatible with adenopathic conglomerate was observed. Due to hematological disease progression, treatment with dexamethasone 8 mg every 12 hours was initiated with significant glycemia improvement ( Graphic 1 ). During hospitalization, the patient developed a left lower limb subacute deep venous thrombosis and anticoagulation treatment with sodium heparin was administered. In July 2015, the patient passed away after a hemodynamic shock probably due to pulmonary tromboembolism.

Although we were unable to measure IGF-2 to confirm the diagnosis of non-islet hypoglycemic cell tumor (NICHT), biochemical data mentioned above and the patient’s improvement after being treated with glucocorticoids confirmed our suspicion. He also developed malignant hypercalcemia, which could mean that both, hypoglycemia and hypercalcemia, were manifestations of paraneoplastic secretion from the same tumor.

## DISCUSSION

Hypercalcemia is defined as an increase in the serum calcium level above the upper limit of normal for a given reference value used in a laboratory ( 6 ).

The differential diagnosis of hypercalcemia includes multiple pathologic entities but is focused primarily on primary hyperparathyroidism and hypercalcemia of malignancy given the highest prevalence of these etiologies ( 6 ) accounting for more than 90% of cases. Once hypercalcemia is confirmed, the next step is serum PTH measurement.

In presence of low serum PTH concentrations (< 20 pg/mL), PTH-rP and vitamin D metabolites should be measured to assess for malignant hypercalcemia and vitamin D intoxication. If these are also low, another source for the hypercalcemia must be considered. Additional laboratory data including serum protein electrophoresis, for possible multiple myeloma, and thyroid-stimulating hormone (TSH), will often lead to the correct diagnosis ( 7 ).

Cancer-induced hypercalcemia (CIH) occurs in 5% to 30% of patients with cancer during the disease course. Depending on the type of tumor CIH represents the most common paraneoplastic syndrome. Lung and breast cancer, and myeloma have the highest incidence of CIH, accounting for more than 50% with a mean survival rate of 2 to 3 months ( 7 ).

Among patients with renal cell carcinoma, CIH is the most common paraneoplastic syndrome affecting between 13 to 20 percent of patients, while in patients with non-Hodgkin lymphoma the incidence achieves 1 to 4 percent, except high-grade lymphomas such as diffuse large B cell lymphoma where up to 30 percent of patients may be affected. Unlike what happens in the RCC, hypercalcemia in lymphoma is primarily mediated by secretion of calcitriol ( 8 ).

Hypercalcemia associated with cancer can be classified into four types: 1) local osteolytic hypercalcemia, results from the marked increase of osteoclastic bone resorption in areas surrounding the malignant cells within the marrow space, 2) humoral hypercalcemia of malignancy (HHM) which is caused by systemic secretion of PTHrP, and causes increased bone resorption and enhances calcium renal retention 3) 1,25-dihydroxyvitamin D secretion by some lymphomas and 4) ectopic secretion of authentic PTH, a rare cause of hypercalcemia ( 6 - 9 ). HHM is the most common cause associated with cancer ( 6 - 9 , 10 ).

PTHrP shares an homology of 60% in its terminal region with PTH and it can stimulate the same Type PTH/PTHrP receptor expressed in bone and kidney, mimicking the action of PTH by stimulating bone turnover through up-regulation of the expression of the receptor activator of nuclear factor-β k ligand (RANKL), and renal calcium reabsorption ( 9 ).

The optimal treatment for CIH depends on its severity and etiology. General measures include exclusion of all calcium intake and hydrosaline replenishment ( 3 ), patients with hypercalcemia are dehydrated by default due to poor oral intake secondary to nausea, vomiting, altered mental status, and hypercalcemia-induced nephrogenic diabetes insipidus ( 6 ). Once the non-pharmacological measures of the treatment of hypercalcemia are taken, and even simultaneously, pharmacological measures can be implemented ( 3 ).

Drug therapies include: bisphosphonates, loop diuretics, calcitonin, glucocorticoids, cinacalcet and monoclonal antibodies ( 6 - 8 - 10 ).

Regarding the presented case, due to the impossibility of measuring PTHrP and 1,25-dihydroxyvitamin D in our country, we reached the diagnosis of malignant hypercalcemia secondary to PTHrP by confirming hypercalcemia with low PTH levels considering oncological disease. As he was refractory to intravenous hydration and his renal function was impaired, which contraindicated the use of intravenous bisphosphonates, subcutaneous denosumab was prescribed with a full response from the patient.

Hypoglycemia is defined as blood glucose below 55 mg/dL, in patients without diabetes, associated with signs and symptoms of hypoglycemia, symptoms relief with oral intake of glucose or food (Whipple’s Triad). The most common causes are related to diabetic treatments, endocrine deficiencies and, less frequently, NICHT ( 4 ).

NICHT consists of a rare syndrome characterized by the presence of a solid tumor and severe fasting hypoglycemia determined by an insulin-independent pathway ( 11 ). In 1929, Nadler and Wolfer described a case of hypoglycemia associated with liver carcinoma for the first time ( 11 ), and Daughaday and cols *.* were the first to demonstrate the presence of IGF-2 in a hypoglycemia-producing tumor ( 12 ).

Data on the exact incidence and prevalence of NICTH are not available. It has been estimated that NICTH is four times less common than insulinoma. The renal tumor represents 1% of NICHT ( 4 ). NICTH is thought to be a fasting hypoglycemia characterized by: 1) diminished hepatic glucose production due to inhibition of glycogenolysis and gluconeogenesis 2) diminished lipolysis, 3) increased peripheral glucose consumption. These phenomena point to an enhanced insulin-like activity caused by the action of IGF-2, which competes for insulin and IGF-1 receptors, and inhibits the secretion of growth hormone (GH), inhibiting IGF-1 and IGFBP3 hepatic production ( 4 ).

IGF- 2 presents great structural similarity with proinsulin and exists in three different forms. In NICTH, it is distributed as follows: a) free IGF-2 represents less than 1% and has a half- life of approximately 10 minutes; b) 20-30% is bound to an IGFBP with a half-life of 30 minutes; c) a complex binary form of higher molecular weight called “big IGF 2” or prohormone which corresponds to 70-80% ( 4 - 13 ).

NICHT diagnosis is performed by the Whipple’s triad, low levels of insulin, C-peptide, GH, IGF-1 and IGFBP-3, and high levels of IGF-2 or big IGF-2, or a ration of IGF-2: IGF-1 greater than 3. The rapid response to glucocorticoids is another parameter to be considered ( 14 , 15 ).

One limitation was that our country does not have the resources to determine serum IGF 2. However, we emphasize that out of a series of 44 patients with NICHT, 13 were Big IGF-2 negative ( 11 ), and as in our case, 78 patients of the series published by Fukuda and cols. showed very low levels of insulin, IGF 1, IGFBP3 and GH, which returned to their normal levels after the tumor was removed ( 15 ).

Most tumors present a single paraneoplastic manifestation, being unusual the coexistence of two or more of them. That situation is fairly frequent in hepatocellular carcinoma where the presence of paraneoplastic syndromes remarkably reduces the survival of patients with HCC ( 16 , 17 ). However, these associations have not been previously described in renal tumors.

In the presence of a common paraneoplastic manifestation as hypercalcemia, other expressions, such as hypoglycemia, should be searched for. Paraneoplastic syndromes in general represent the sign of a more aggressive cancer and poor prognosis. Therefore, they are of clinical significance and deserve further study to provide a better treatment strategy.
